# Stanniocalcin2, A Promising New Target for Identifying Patients with Stroke/Ictus

**DOI:** 10.3390/ijms26209999

**Published:** 2025-10-14

**Authors:** Nuria Bermejo, José Javier López, Alejandro Berna-Erro, Esperanza Fernández, Antonio Jesús Corbacho, Maria Teresa Vázquez, Maria Purificación Granados, Pedro Cosme Redondo

**Affiliations:** 1 Department of Hematology, Hospital San Pedro de Alcántara, 10005 Cáceres, Spain; nuriabeuve@icloud.com; 2 Department of Physiology, University of Extremadura, 10003 Cáceres, Spain; 3 Department of Biochemistry and Molecular Biology, University of Miguel Hernández, 03202 Elche, Spain; aberna@umh.es; 4 Blood Donation Center, Extremadura County Health Service, 06800 Mérida, Spain; esperanza.fernandez@salud-juntaex.es (E.F.); antonio.corbacho@salud-juntaex.es (A.J.C.); mariateresa.vazquez@salud-juntaex.es (M.T.V.); 5 Pharmacy Unit, Extremadura County Health Service, 10004 Cáceres, Spain; mpgc77@hotmail.com

**Keywords:** STC2, stroke, thrombotic patients, Ca^2+^ homeostasis

## Abstract

STC2 (stanniocalcin 2) controls calcium (Ca^2+^) homeostasis in human platelets and other cell lines. The regulation of intracellular Ca^2+^ homeostasis is crucial for platelet activation; thus, the alteration in intracellular Ca^2+^ concentration or the mechanism involved in its regulation has been proposed to underlie some thrombotic disorders. Our previous studies evidenced that the knockdown of STC2 altered murine platelet activation; furthermore, a reduction in STC2 expression resulted in enhanced Ca^2+^ homeostasis in diabetic patients and, therefore, would contribute to the prothrombotic condition as a hallmark of diabetes mellitus type 2 (DM2). In this study, we examine a possible link between the expression of stanniocalcins (STCs) and different thrombotic events in humans. The expression of STCs was determined by Western blotting (WB); meanwhile, the analysis of protein interaction and phosphorylation was performed by completing a previous immunoprecipitation protocol (IP) of the proteins of interest. Thus, our results from patients with stroke/ictus presented a clear reduction in STC2 expression in their platelets, finding less STC2 content in the youngest thrombotic patients. Furthermore, acetyl-salicylic acid (ASA) administration reversed the decrease in the expression of STC2 in patients who did not suffer additional thrombotic episodes, as evidenced by the longitudinal analysis of up to 10 years of follow-up. Additionally, the increase in STC2 phosphorylation at the serine residues revealed increased activity of STC2 in thrombotic patients. Finally, we suggest that store-operated Ca^2+^ entry (SOCE) is over-activated in patients suffering from stroke/ictus, as revealed by the increase in the STIM1/Orai1 interaction found under resting conditions and, further, because MEG-01 cells transfected with siRNA STC2 to evoke artificial reduction in the STC2 expression presented an increased SOCE with respect to the control cells transfected with siRNA A. Conversely, the expression of the non-capacitative Ca^2+^ channels, Orai3 and TRPC6, was found to be reduced in patients with stroke. Altogether, our data allow us to conclude that STC2 represents a promising marker of stroke/ictus in thrombotic patients.

## 1. Introduction

Thrombotic diseases comprise a wide variety of illnesses of different molecular, genetic, and cellular backgrounds, mostly affecting the heart, brain, lungs, or kidneys, and hence, they have a deleterious effect on the human body. These circulatory incidents often occur in smaller vessels where blood flow is restricted due to the appearance of pathological blood clots known as thrombosis. It is worth mentioning that according to a WHO report (2020), cardiovascular stroke and brain stroke are the leading causes of non-transmissible deaths in the entire world population. Among thrombotic illnesses, cardiac stroke is at the top of the list; meanwhile, brain stroke (ictus) is in third place in high-income countries, being the cause of up to 2.5 million deaths (WHO, 2020 [1]). Hence, for many countries, it is compulsory to discover new and precise molecular markers as well as protein targets for developing new drugs that may prevent thrombotic events. Despite the fact that thrombosis has been generally described as a blood clot evoked by platelet activation, nowadays, we know that it is driven by different hemostatic disorders, such as micro-thrombosis, fibrin clot disease, and many others [2]. Platelet hyper-activation is a crucial step during the development of thrombotic events; therefore, the analysis of the physiological and molecular alterations that may occur in platelets of the thrombotic patients is relevant for understanding this disease. The changes in intracellular Ca^2+^ homeostasis ([Ca^2+^]_i_) or signaling pathways that trigger changes in [Ca^2+^]_i_ have been reported to be crucial for regulating platelet function. In this sense, in the last decade of the past century, elevated Ca^2+^ homeostasis has been reported in platelets from thrombotic patients [3]. Interestingly, patients suffering from venous thrombosis (DVT) present a reduced [Ca^2+^]_i_ than patients with cardiovascular accident or occlusive vascular diseases [3]. More recently, it has been described that platelets from patients with arteriosclerosis exhibit elevated [Ca^2+^]_i_ with respect to platelets from healthy subjects. However, the changes in [Ca^2+^]_i_ evoked by stimulating the platelets with the physiological platelet agonist, thrombin (Thr), were smaller in those drawn from thrombotic patients than in those from healthy subjects. Thus, these results suggest the existence of chronic activation of platelets in thrombotic patients [4]. On the other hand, in 2006, it was identified that stromal interaction molecule 1 (STIM1) and its target plasma membrane Ca^2+^ channel, Orai1, are both involved in the activation of store-operated Ca^2+^ entry (SOCE) in human platelets, which is also known as capacitative Ca^2+^ entry (CCE), but the latter may also involve other Ca^2+^ channels in addition to Orai1, like TRPC1 [5,6,7]. This particular Ca^2+^ entry mechanism has been reported to be altered in patients suffering from thrombotic events and is particularly relevant to those suffering from Stormorken’s syndrome [8,9]. These patients express a mutation in STIM1 that leads to elevated Ca^2+^ entry through Orai1, resulting in enhanced aggregation. In addition, several STIM1/Orai1 regulators have been described over the last decade, and subsequently, they were also described to be crucial for platelet activation. Further, they can be deregulated in hyper-reactive platelets. For example, SARAF (a STIM1 inhibitor) has been found to be overexpressed in neonatal platelets, leading to a reduced Thr-dependent Ca^2+^ entry activation [10,11,12]. Thus, neonatal platelets presented increased SOCE instead because of the presence of a calpain-dependent cleavage domain within the STIM1 structure that would prevent its interaction with SARAF [12,13,14,15,16].

Finally, SOCE is also downregulated by stanniocalcin 2 (STC2), as evidenced in MEF cells [17], and it is one of the two isoforms described in humans (STC1 and STC2). STC2 regulates intracellular phosphate and the Ca^2+^ concentration in mammals, among others, which are intracellular signaling mechanisms [18,19]. We have previously analyzed the alterations in murine platelets from STC2 knockdown mice, which exhibited enhanced Ca^2+^ entry and hyperaggregability with respect to platelets from WT mice [20]. In addition, SOCE was not altered, but elevated expression of non-SOCE channels, such as Orai3, was found in those platelets [20]. Recently, we also demonstrated reduced STC2 expression in platelets from type 2 diabetes mellitus patients (DM2) [21]. These initial observations were confirmed by other groups that found an alteration in the plasma concentration of STC2 and pregnancy-associated plasma protein A (also known as pappalysin-1 or PAPP-A or PAPPA1) in DM2 patients suffering from cardiovascular diseases [22]. It is worth mentioning that it has been demonstrated that STC2 is an inhibitor of PAPP-A and, thus, STC2 is also involved in the regulation of the insulin growth factor (IGF) axis. The release of STC2 from PAPP-A leads to liberation of IGF1 from IGF-binding proteins (IGFBPs), particularly by enzymatic cleavage of IGFPB4, thus activating this intracellular pathway [22,23]. Taking all this information into account, we aim to elucidate a possible link between the alteration in the expression of STCs and the possible platelet alteration found in thrombotic patients.

## 2. Results

### 2.1. Expression of STC1 and STC2 in Thrombotic Patients

**Figure 1:**
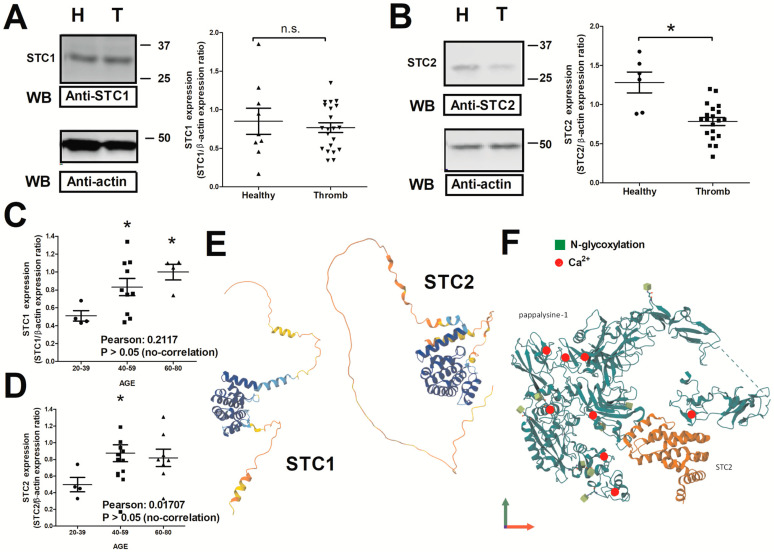
Analysis of the expression of STC1 and STC2 in platelets from thrombotic patients (T) vs. healthy subjects (H). (**A**,**B**). Platelet samples drawn from either healthy subjects or thrombotic patients were immediately isolated by performing differential centrifugation, and they were subsequently lysed in Laemmli’s buffer (LB). Dot-plots represent the values of the protein expression analyzed by WB using anti-STC1 (n = 22) and anti-STC2 antibodies (n = 25). The membranes were reprobed with an anti-β-actin antibody that was considered as protein loading control. (**C**,**D**) Dot-plots show the intragroup comparative analysis considering age and the STC1/STC2 expression values. (**E**) AlphaFold^®^-based structures of STC1 and STC2 are presented for comparison purposes (https://alphafold.ebi.ac.uk/entry/O76061; accessed on 1 May 2025). (**F**) Representation of the AlphaFold^®^ structure of PAPP-A is shown while it interacts with STC2 *: represent *p* > 0.05; Kruskal–Wallis was used for data analysis. n.s.: not significant.

Western Blotting (WB) analysis of the expression of STC1 and STC2 in platelets is presented in Figure 1. The expression of STC1 did not result in a statistically significant change in patients suffering from thrombotic events with respect to healthy subjects. Conversely, we observed a significant reduction in the STC2 values in the group of thrombotic patients (Figure 1A,B; n = 18 & 30, respectively; *p* < 0.05 with respect to the values found in 6–10 healthy subjects). Interestingly, a deeper analysis of the expression values of STC1 and STC2 evidenced a possible link between age and the expression of both proteins; thus, older thrombotic patients present higher protein concentration, but the correlation analysis did not corroborate this hypothesis (Figure 1C,D; Pearson’s correlation coefficient and statistical values are indicated). Additionally, different molecular structures have been proposed for STC1 and STC2 (as shown in Figure 1E), which may explain why both proteins are involved in different cellular signaling pathways. In fact, STC2 participates as a negative regulator of PAPP-A by generating a dimer before docking into the regulatory pocket of PAPP-A. As shown, STC2 contains eight Ca^2+^-binding regions that allow it to feel [Ca^2+^]_i_ changes (see Figure 1F).

**Figure 2:**
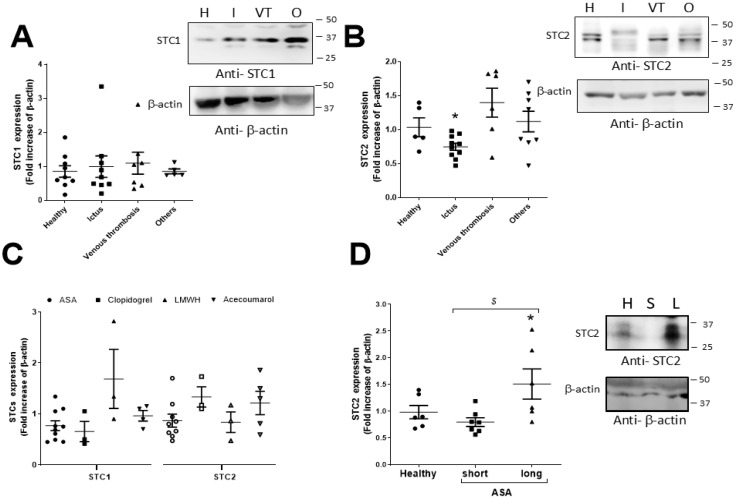
Analysis of the changes in STC expression according to thrombotic diseases and the drugs administered to patients. (**A)** STC1 and STC2 **(B**) expression values were determined by WB, and they were distributed in different population subgroups according to the diagnosed thrombotic illnesses (n = 6–8; healthy (H): 8; ictus (I): 8; venous thrombosis (VT): 6; others (O): 6). (**C**) Expression values of both STCs were reanalyzed according to the different drugs prescribed to thrombotic patients (n = 3–10; ASA: 10; clopidogrel: 3; LMWH: 3; acenocoumarol: 5). (**D**) Platelets were drawn from healthy subjects (H, n = 6) and patients suffering from stroke/ictus that were medicated either with the different anti-thrombotic drugs (ictus; n = 9), with 100 mg of ASA daily for 1 year (short-time period: S; n = 7) and treated with ASA (100 mg) for 10 years (long-time period: L; n = 6–8). Platelet lysates were analyzed by WB using an anti-STC2 antibody, and the reprobing of membranes was performed with an anti-β-acting antibody as described in Materials and Methods. WB images are representative of 3–4 independent experiments. *: *p* < 0.05 with respect to healthy subjects; *$*: *p* < 0.05 with respect to patients with stroke and treated with ASA for short time periods. Kruskal–Wallis test and Dunn’s post-test were used for data analysis.

To deeply analyze the possible role of STCs in the development of thrombotic diseases, we confronted the expression values of both STCs with the different types of illnesses diagnosed in our thrombotic cohorts. As depicted in Figure 2A,B, the analysis of the expression values of STC1 did not indicate statistical differences among the types of the thrombotic incident considered; however, the expression of STC2 was altered in thrombotic patients with stroke/ictus (I) and deep venous thrombosis (VT), but only patients suffering from stroke/ictus presented a statistically significant reduction in the expression values of STC2 with respect to those observed in healthy subjects (Figure 2B; I, 0.79 ± 0.06, *p* < 0.05; n = 16 vs. H, 1.0 ± 0.13, n = 15–30, respectively).

Finally, we analyzed the possible correlation between the type of drug administered at the time of first evaluation and the changes in the expression of both STCs. It is worth mentioning that most of the patients were analyzed for the first time during the initial 6 months after the first thrombotic episode (so-called “sort administration”), but we did not find statistical differences between the treatment administered to the patients and STC2 or STC1 expression values.

Nonetheless, the patients medicated with a daily dose of acetyl salicylic acid (100 mg of ASA) and low-molecular-weight heparin (LMWH) presented reduced STC2 expression values; meanwhile, those under medication with clopidogrel or acenocoumarol exhibited the highest STC2 expression values (Figure 2C). Interestingly, STC2 values were not significantly affected by the acute administrations of antithrombotic agents (Figure 2C). However, long-term administration of ASA of up to 10 years was able to reverse the reduction in the STC2 expression found in patients suffering from stroke (Figure 2D; n = 5, *p* < 0.05).

### 2.2. Analysis of Clinical and Demographic Variables That May Correlate with the STC2 Expression Values in Thrombotic Patients

**Figure 3:**
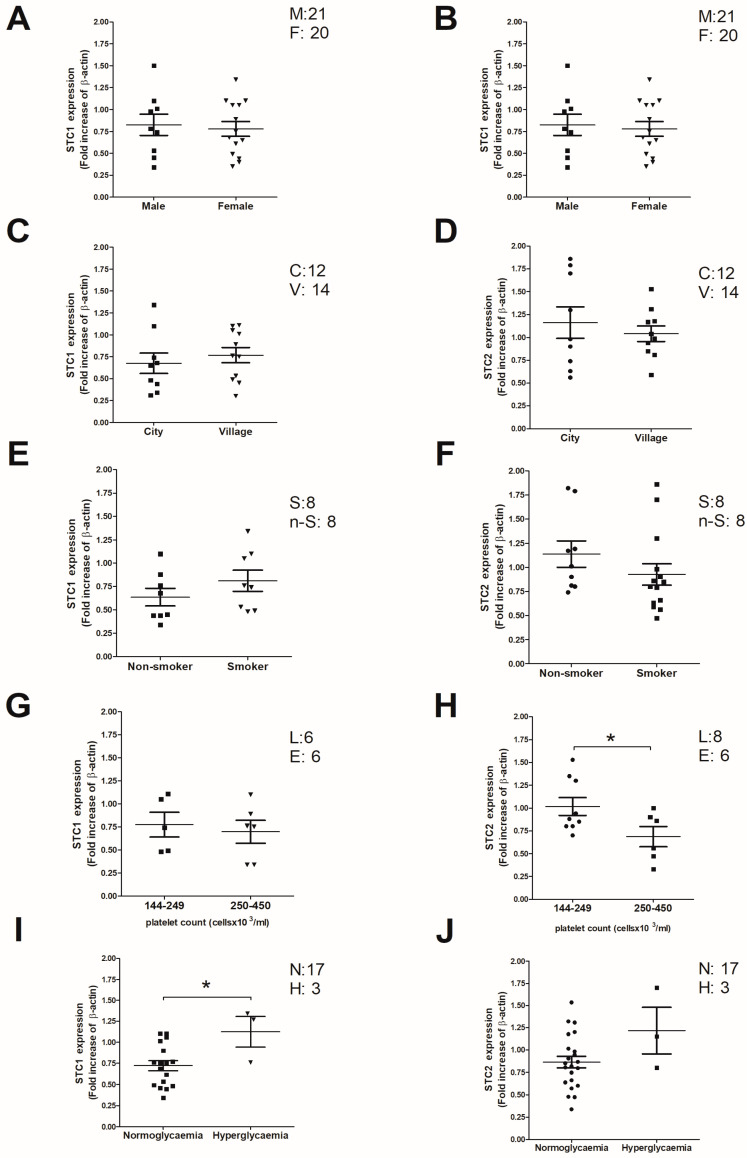
Analysis of possible patient demographic and clinical variables linked with STC1 and STC2 expression. (**A**,**C**,**E**,**G**,**I**) Platelets from thrombotic patients were isolated and lysed for analysis by WB with an anti-STC1 antibody or with an anti-STC2 antibody (**B**,**D**,**F**,**H**,**J**) as described in Materials and Methods. *: *p* < 0.05 with respect to normoglycemic thrombotic patients and patients with normal to high platelet count. Mann–Whitney U-test was used for statistical analysis. Numbers on the right-hand side of the dot-chart indicate the subjects included in each subgroup that are identified according to the following nomenclature: M: male; F: female; C: cities; V: villages; S: smoker; n-S: non-smoker; L: low platelet count; E: high platelet count; N: normoglucemia; H: hyperglycemia.

The appearance of an illness has different components that are circumscribed by genetic predisposition, which results in changes in the expression of proteins, as well as in their possible mutation. Further, this genetic predisposition may be aggravated by environmental conditions. So, we analyzed a possible correlation between the STC2 expression values and environmental or other variables, like gender or the surrounding living environment (village vs. cities), but we did not find significant differences among the patient subgroups independently of the STC isoform considered (Figure 3A–D). Additionally, another variable considered was smoking habits, which seem to be correlated with a reduction in the expression values of STC2, but the values found in the correlation analysis did not reach statistical significance. STC1 did not correlate with smoking habits (Figure 3E,F).

Conversely, a reduction in STC2 expression values was observed in thrombotic patients with a medium/high platelet count with respect to those where platelet count was below or around the limit and hence could be diagnosed as suffering from thrombocytopenia (less than 150 × 10^3^ platelet/μL; *p* < 0.05); meanwhile, STC1 was not found to be modified in these patients (Figure 3G,H indicate STC1 and STC2 expression values, respectively) [24]. Finally, the expression of STC1 is different in patients suffering from comorbidities like hyperglycemia, but in these hyperglycemic patients, STC2 was not significantly modified (Figure 3I,J, which indicate the expression values of STC1 and STC2, respectively); nonetheless, more patients suffering from both comorbidities are required to finally confirm this observation.

### 2.3. Analysis of STC2 Post-Translational Modification by Phosphorylation in Patients Suffering from Stroke

**Figure 4:**
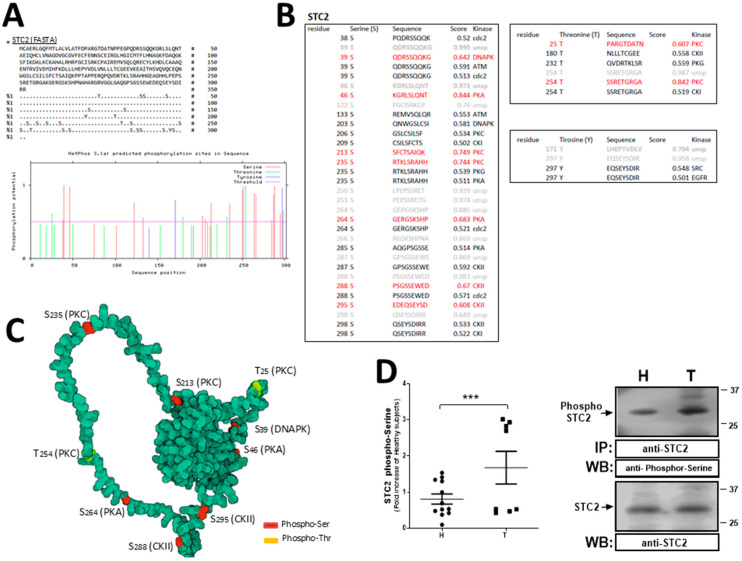
Analysis of the STC2 phosphorylation at serine residues in platelets from thrombotic patients. (**A**–**C**) The “In silico” analysis of the post-translational modifications of STC2 by phosphorylation was performed by using NetPhos 3.1 (https://services.healthtech.dtu.dk/services/NetPhos--3.1/; accessed on 1 May 2025). (**A**) The FASTA sequence is shown, highlighting the residues of interest; meanwhile, tables in (**B**,**C**) represent the phosphorylation score obtained using NetPhos 3.1 software and include the possible kinases that are positively identified as targeting those residues. (**C**) AlphaFold^®^-based structure of STC2 is represented, highlighting residues that presented the highest scores (https://alphafold.ebi.ac.uk; accessed on 1 May 2025). (**D**) Platelets from thrombotic patients (T) and healthy subjects (H) were isolated and, subsequently, the proteins were immunoprecipitated using an anti-STC2 antibody. The samples were solved by WB using an anti-phospho-serine antibody, and reprobing of the membranes to ascertain the protein loading control was performed with an anti-STC2 antibody as described in Materials and Method section. Dot-plot represents the mean ± S.E.M. of fold-increase with respect to healthy volunteers and considering 8 to 10 independent IPs. Images are representative of four to six different WB. ***: *p* < 0.001 with respect to healthy subjects. Mann–Whitney U-test was used for statistical analysis.

Protein activity can be altered by post-translational modifications like phosphorylation, glycosylation, and ubiquitination. We initially performed an “in silico” analysis of the possible residues that could be suitable for phosphorylation in STC2 (Figure 4). Using free NetPhos 3.1 software, we analyzed the sequence of STC2 by focusing on the phosphorylation of the serine (S), tyrosine (Y), and threonine (T) residues. As a result, we found that STC2 can mainly be phosphorylated at serine residues (Figure 4A), where PKA (Ser46 and Ser246) and PKC (Ser213 and Ser235) are the kinases with the highest scores (Figure 4A–C; red: sequences obtaining the highest scores of already-identified kinases; gray: residues with the highest score that are targeted by an unknown kinase) and, further, the ectokinase casein kinase II (CKII) that phosphorylates STC2 at Ser288 and Ser295 (Figure 4B,C). Additionally, PKC also phosphorylates STC2 at threonine residues like Thr25 and Thr254 (with the latter achieving the highest score) (Figure 4B,C). Finally, tyrosine phosphorylation of STC2 could be discarded because of the low score obtained, where Tyr254 is the residue phosphorylated by Src and EGFR with the highest probability (Figure 4B). According to this “in silico” analysis, we performed a set of experiments to confirm that STC2 is actually phosphorylated at serine residues and whether this phosphorylation may be modified in thrombotic patients. Therefore, platelets from healthy volunteers (H) and thrombotic patients (T) were isolated and lysed under resting conditions. Immunoprecipitation of platelet lysates using an anti-STC2 antibody and, subsequently, analysis by WB with an anti-phospho-serine antibody revealed a significant 1.6 ± 0.14 fold increase in STC2 phosphorylation at serine residues with respect to the values found in healthy volunteers (Figure 4D; *p* < 0.05, n = 8–11).

Interestingly, the glycosylation of STC2 has also been reported in the literature, which was also confirmed here by incubating the platelet lysates from healthy subjects or MEG-01 and HEK293 cell lysates overnight with PNGase F (Supplementary Figure S1D.1). PNGase F incubation modified the pattern of bands for STC2, which was much more evident in human platelets and MEG-01 cells, where an additional band (25–30 kDa) appeared in the image of WB for STC2; this additional band would migrate bellow the normal band at ~33–35 kDa. Conversely, in HEK293 cells, protein migration upon PNGase F treatment was less evident, appearing as a small band just below the canonical one (~30 kDa) and in parallel with a reduction in the ~62 kDa band that would be indicative of the presence of a possible glycosylated STC2 dimer. Additionally, other alternative bands appeared at 50 and over 75 kDa that were particularly evident in human platelet samples. However, they were not considered in the present analysis because they may have been derived due to unspecific recognition of the secondary antibody, as demonstrated in Supplementary Figure S1D.2, where membranes were only exposed to the secondary HRP-conjugated anti-IgG antibody, and these bands almost disappeared in the samples corresponding to other cell lines.

### 2.4. Regulation of the Capacitative Ca^2+^ Channels by STC2 in Patients Suffering from Stroke

**Figure 5:**
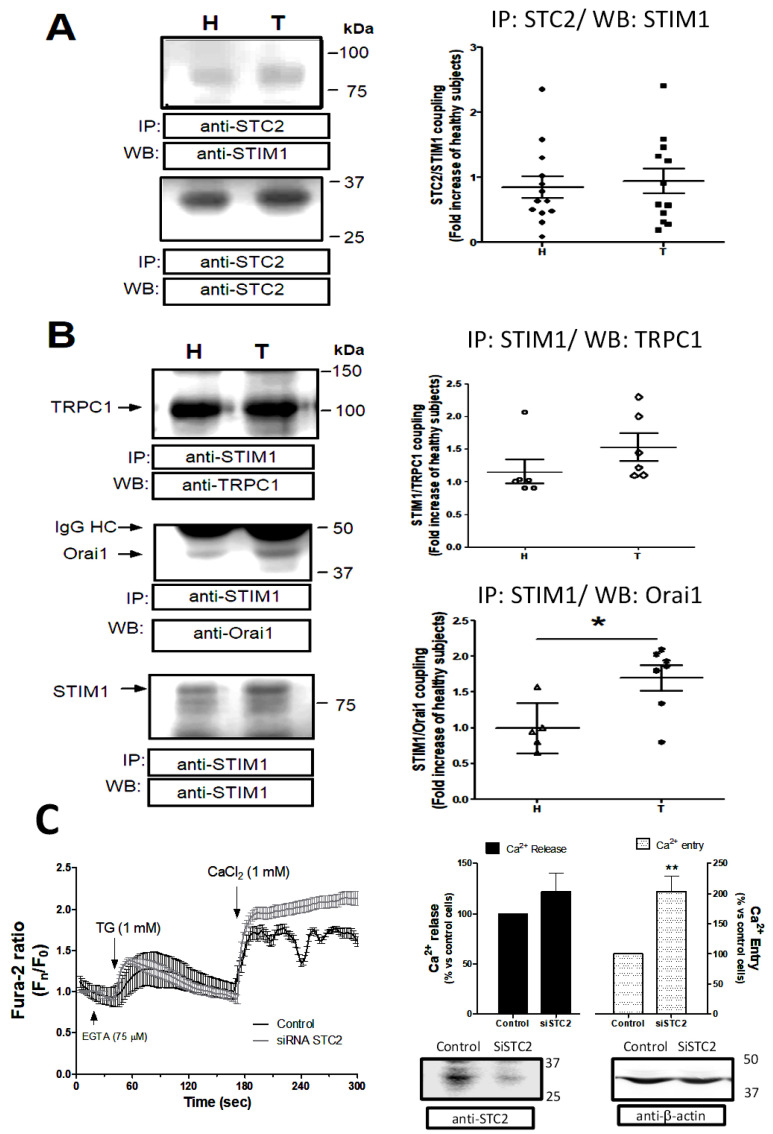
SOCE alteration in platelets from thrombotic patients. Platelets from thrombotic patients (T) and healthy subjects (H) were isolated and lysed with ice-cold NP40 buffer. Then, the platelet samples were immunoprecipitated using 2 μg/mL of either an anti-STIM1 or anti-STC2 antibody and beads of agarose. Subsequently, the protein interaction was analyzed by WB using an anti-STIM1 antibody (**A**) or anti-TRPC1 and anti-Orai1 antibodies as indicated (**B**). Membranes were reprobed with an anti-STC2 (**A**) or anti-STIM1 (**B**) antibody, which were used as protein loading controls, respectively. Dot-plots represent mean ± S.E.M. of fold increase with respect to healthy volunteers and considering 5–6 independent IPs. Images are representative of four different WB. (**C**) MEG-01 cells were transfected with siRNA STC2 or siRNA A (control), and, subsequently, the evaluation of the changes in SOCE evoked by TG was analyzed by considering up to 60–70 cells from up to 4 independent transfections, as described in Materials and Methods. The bottom images demonstrate that STC2 has been silenced, and they are representative of 4 independent transfections and WB with an anti-STC2 antibody, and an anti-β-actin antibody was used as protein loading control. *, **: *p* < 0.05 and < 0.001 with respect to healthy subjects. Mann–Whitney U-test was used for statistical analysis.

As described in the Introduction section, STC2 has been described as a multifunctional protein that is capable of regulating several intracellular mechanisms, such as hypoxia downstream of HIF1, acting as a heat shock protein [25,26]. Furthermore, STC2 interacts with metal ions or regulates phosphate and Ca^2+^ homeostasis [17]. Regarding the latter function, previous studies have described that STC2 is a negative modulator of SOCE, and considering that SOCE is a crucial mechanism for platelets’ function, we analyzed the coupling between STC2 and STIM1. Thus, as depicted in Figure 5A, STC2 coimmunoprecipitated with STIM1, even in resting platelets, but no statistical differences were found when we considered all the analyzed platelet samples from thrombotic patients with respect to the samples from healthy subjects (*p* > 0.05; n = 8).

**Figure 6:**
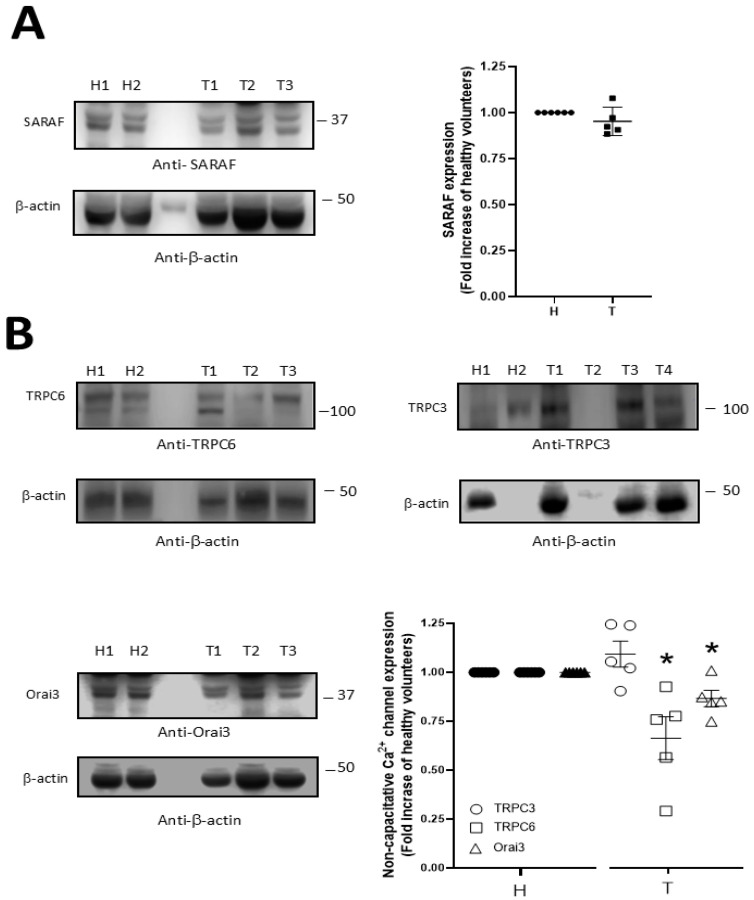
Expression of SARAF and other Ca^2+^ channels in thrombotic patients. Platelets from thrombotic patients (T) and healthy subjects (H) were isolated and lysed with Laemmli’s buffer. Next day, platelet samples were analyzed by WB using an anti-SARAF antibody (**A**) and anti-TRPC6, TRPC3, and Orai3 antibodies as indicated (**B**). The reprobing of the membranes was performed using an anti-β-actin antibody as protein loading control. Dot-plots represent mean ± S.E.M. of fold increase with respect to healthy volunteers and considering 5-6 independent platelet samples. Images are representative of three different WB. *: *p* < 0.05 with respect to healthy subjects. Mann–Whitney U-test was used for statistical analysis.

Interestingly, platelets under resting conditions presented clear immunoprecipitation between STIM1 and Orai1, and further, a significant increase in the association of both proteins was observed in platelets from thrombotic patients with respect to healthy subjects (1.8 ± 0.14 fold increase; *p* < 0.05, n = 5; Figure 5B), which may be indicative that SOCE is preactivated in these patients. Conversely, although TRPC1/STIM1 coupling was found to be elevated in platelets from thrombotic patients, no statistical difference resulted from the comparison between the thrombotic patients and healthy subjects (1.5 ± 0.3 fold increase; *p* > 0.05, n = 5; Figure 5B). As depicted in Figure 5C, MEG-01 cells transfected with SiRNA STC2 did not present alteration in TG-evoked Ca^2+^ release with respect to control cells transfected with SiRNA A (control cells); meanwhile, a significant increase in the TG-evoked SOCE was observed in the cells lacking STC2 (205 ± 19%; n = 4, *p* < 0.001). Therefore, we demonstrated that in the platelet lineage, reduced STC2 expression would lead to increased SOCE activation. On the other hand, we did not find changes in the expression of the main regulator of SOCE, SARAF, as revealed by performing the analysis using WB and using platelets drawn from both groups analyzed here (see Figure 6A; *p* > 0.05, n = 7).

Finally, in a previous study using platelets drawn from STC2 KO mice, we found increased Ca^2+^ entry because of enhanced expression and activation of non-capacitative channels; thus, we evaluated the expression of other Ca^2+^ channels that are considered non-capacitative Ca^2+^ channels (Orai3, TRPC3, and TRPC6) in platelets from thrombotic patients. As shown in Figure 6B, the expression of TRPC3 was not altered in the platelets of both groups; meanwhile, the expression of TRPC6 and Orai3 was reduced by 13.3 ± 4% and 24.1 ± 15.9% in thrombotic patients with respect to healthy subjects, respectively (*p* < 0.05; n = 6–8).

## 3. Discussion

STCs have been described as proteins with high similarity to the hormones isolated from the corpuscles of Stannius found in fish [27]. The first described STC was called teleocalcin or hypocalcin because it controls Ca^2+^ homeostasis at the body level, but scientists soon discovered that they were structurally different from the parathyroid hormone (PTH) [28,29]. Nowadays, the involvement of STC1 and STC2 in cell and body physiology has changed as a result of evolution; the two members of the STC family display different roles as well as different protein structures. Thus, STC1 regulates glucose metabolism or oxidative stress; meanwhile, STC2 controls Ca^2+^ and phosphate metabolism, as well as other metal ions. However, the role of both proteins may converge in diseases like cancer linked with reproductive organs, where both isoforms contribute to worsening the life span of the patients by favoring cancer progression. In line with this, STC1 participates in the notch1 axis or in the PKB/ATK/mTOR axis [30,31,32]; meanwhile, STC2 is overexpressed in several types of cancer, including breast, ovarian, and cervical cancers. Interestingly, STC2 is also altered in other reproductive malignancies like eclampsia during pregnancy [33,34]. Regarding the pathologies that may compromise blood circulation, a recent investigation claimed that STC1 reduces the effects associated with myocardial ischemia–reperfusion injury [35] and, further, the activation of PAR1 leads to STC1 upregulation, which may act as cytoprotection in the case of thromboinflammatory injury [36]. On the other hand, STC2 was reported to be involved in chronic thromboembolic pulmonary hypertension downstream of TGF-induced protein [37]. In previous studies using platelets of STC2 KO mice, we found enhanced platelet activation in response to Thr and adenosine-3’,5’-diphosphate (ADP), and in “in vivo” experiments, we also observed shorter tail bleeding, which allowed us to conclude that STC2 has a clear regulatory role in platelet function [20].

Later on, we described reduced expression of STC2 in DM2 patients, who often exhibit a prothrombotic status that underlies retinopathy and diabetic feet, among other hallmarks [21,38]. Here, we also described for the very first time that in the platelet lineage (MEG-01 cells and platelets), a reduction in STC2 results in increased SOCE. In addition, we did not find changes in the expression of STC1 in platelets from thrombotic patients, but we confirmed that STC2 is involved in the regulation of platelet function, because its expression is statistically downregulated in thrombotic patients and, therefore, closing the circle between our initial observations performed on murine platelets STC2 Ko [20] and this later study performed on thrombotic patients. However, the link between STC2 and thrombosis has to be much more complex, because not all thrombotic patients presented a reduction in STC2 expression, such as those suffering from venous thrombosis, who did not present significant changes, and even a small number of these patients exhibited STC2 levels above those found in healthy subjects. Interestingly, VT may appear as a complication of previous stroke/ictus conditions and, thus, STC2 may represent a possible molecular tool to identify and differentiate the origin of both types of thrombotic events. Unfortunately, we could not find a statistical correlation between patients who presented altered pro-thrombotic markers and reduced STC2 platelet expression, but this controversy may be explained because these patients were immediately medicated with the antiplatelet therapy of choice (often ASA) and discharged from the emergency unit upon reaching stabilization of these markers to regular values.

Administration of ASA for short time periods (less than a year of medication with 100 mg on a daily basis) did not statistically modify the STC2 expression values observed in the stroke/ictus patient group, despite reduced STC2 expression being found in comparison to other drugs (Figure 2B). In this study, we experimentally evidenced that ASA (100 mg daily) reversed the reduction in the STC2 expression values after 10 years of administration (Figure 2C), but we only monitored the STC2 values in patients whose initial clinical conditions or drug administration were not altered in the last decade; otherwise, patients were excluded from the second tests. We are also aware that a main limitation in this study is the fact that some samples were frozen and stored for a long time period (over a decade). This issue must affect healthy samples and samples from patients who were suggested to undergo short ASA administration. These samples are susceptible to being altered due to the prolonged frozen period, and as a result, data regarding long-time exposition with ASA should be carefully interpreted. However, our observations agree with others reporting that ASA administration impairs STIM1/Orai1 interaction, and hence, ASA reduces SOCE in colorectal cancer cells; meanwhile, other nonsteroidal anti-inflammatory drugs, such as sulindac, impair SOCE and STIM1 membrane translocation [39]. Therefore, the effect of ASA on STC2 expression favors the impairment of SOCE activation in thrombotic pathways, which may be added to the well-known anti-aggregation effects of ASA, which is a widely known COX antagonist in platelets. The molecular mechanism through which STC2 expression is altered by COX deserves future investigation, especially considering that COX2 favors STC1 expression in response to arginine vasopressin [40], and miR-146a avoids the expression of COX2, which leads to STC1 overexpression [41]. So, this indicates that both signaling pathways may be somehow interconnected. Finally, a recent study demonstrated the benefit of the intravascular administration of STC2 to recover infarcted areas within the brain of a stroke murine model. The authors claim that STC2 improves stem cell proliferation, which promotes circulation to the infarcted areas of the murine brain [42].

In line with our observations, STC2 is involved in the HIF-1-dependent signaling pathway in cells exposed to hypoxia [25]. Thus, human tissues exposed to prolonged hypoxic conditions should present enhanced STC2 expression in response to oxidative stress; similarly, ER stress, ATP depletion, or therapeutic stress may enhance STC2 expression downstream of BIP/PERK/p-eIF2a, which requires a preactivation step involving ATF-4 and CHOP [43,44]. So, hypoxic tissues derived from a deep venous thrombosis episode present enhanced STC2 expression and activation; meanwhile, patients with stroke/ictus can not undergo a hypoxic episode and therefore depend on the time elapsed from the initial tissue reperfusion, and, finally, the time required to establish an adequate medication may not necessarily alter STC2 expression. But, it is plausible that these patients suffering from stroke exhibit a reduction in STC2 expression. These results allow us to conclude that STC2 may be a good prognostic marker to identify the initial issue underlying the thrombotic events.

Regarding other clinical variables contemplated here, STC2 is an estrogen-responsive gene co-expressed with the estrogen receptor in breast cancer [45,46,47], but according to our results, we cannot rule out that the change in the expression of STC2 is linked with the gender of our patients. On the other hand, considering that a reduction in STC2 expression leads to a hyperthrombotic state, we would expect that patients expressing less STC2 would have a reduced platelet count due to platelet consumption, but this was not the case in our thrombotic population. Similarly, according to our previous studies, hyperglycemic patients should present reduced STC2 values, but these findings were not reproduced in our thrombotic patients [21]. Both discrepancies should be revisited in future investigations by including a larger-scale multicentre analysis. Conversely, we observed an increase in STC1 expression in thrombotic patients suffering hyperglycemia, which may be indicative of the progression of diabetes illness, which is often complicated by platelet hyperactivation and, subsequently, the thrombotic events associated with diabetes [48]. In line with this, overexpression of STC1 in diabetes patients suffering from kidney complications (diabetic-linked kidney disease) would ameliorate further complications due to the protective role of STC1 against oxidative stress and cell apoptosis [49].

Following, we analyzed whether STC2 activation by phosphorylation may be involved in the regulation of intracellular Ca^2+^ homeostasis. Hence, we can conclude that phosphorylation of STC2 at serine residues is clearly elevated in resting thrombotic patients with respect to the healthy population. Increased STC2 phosphorylation was also found in the plasma of patients suffering from breast cancer, and, although both diseases are not related, they may share some key common intracellular proteins [50,51]. Our analysis revealed that STC2 may be targeted by PKA (mainly at S46 and with a lower score at S235, S264, and S285, respectively), PKC (mainly at S213 and with a lower score at S235; T254 and the lowest score at T25) and CKII (at S288 and with a lower score at S295 and S298; meanwhile, also phosphorylates T180). In line with this, in a previous investigation performed on human fibrosarcoma cells, the authors claimed that PKC would be the main kinase that targets STC2 at intracellular levels; meanwhile, STC2 must be secreted out of the cells to be actually phosphorylated by CKII [52]. In future investigations, we will determine whether the phosphorylation values found in STC2 in platelets are derived from STC2 uptake from plasma after being secreted by other cells suffering hypoxia, such as endothelial cells. Here, the WB image presented in Supplementary Figure S1A does not support the idea that STC2 is secreted to plasma upon SOCE activation with TG, and, further, considering that there is no clear presence of STC2 in our plasma samples, the idea of STC2 reuptake from plasma of patients is supported experimentally. Additionally, STC2 glycosylation was also confirmed in human platelets (supplementary Figure S1D.1).

**Figure 7:**
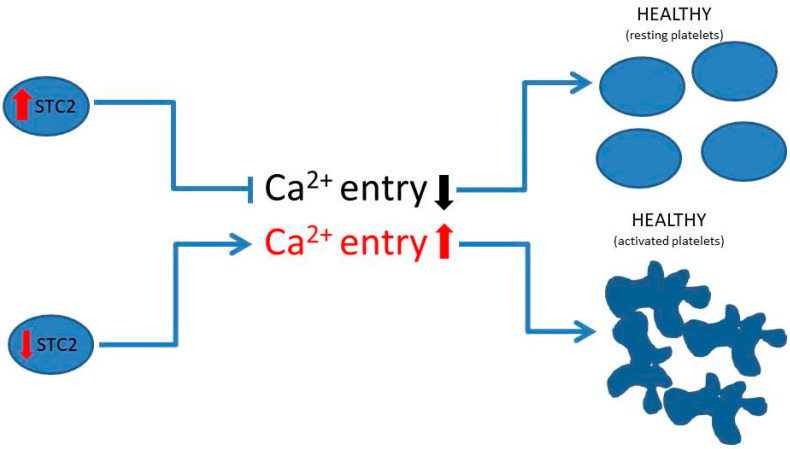
Graphical abstract representing the role of STC2 in the intracellular Ca^2+^ entry in human platelets. In human platelet and MEG-01 cells, a reduced STC2 expression leads to increased intracellular Ca^2+^ homeostasis through an enhanced Ca^2+^ entry (SOCE hyperactivation) that leads to an elevated risk of platelet aggregation. Here, we further describe that this phenomenon is occurring in thrombotic patients suffering from stroke/ictus, but not in other types of thrombotic patients; hence, STC2 may be a relevant marker for this type of illness, but future multicenter studies are required, along with the discovery of specific drugs to modify its expression or function in the platelets of thrombotic patients.

Finally, a clear regulatory role of STC2 in Ca^2+^ homeostasis was demonstrated in fish at the body level; nowadays, STC2′s role in Ca^2+^ homeostasis has been circumscribed at intracellular levels in mammal cells [53]. In this sense, we and other groups have demonstrated a negative role of STC2 in SOCE, which is also confirmed in thrombotic patients and in MEG-01 cells, where STC2 was silenced using SiRNA STC2 (Figure 5C). STC2 interacts with STIM1 in resting platelets, and, although we did not find significant changes in the association between both proteins in platelets from thrombotic patients with respect to healthy subjects, we observed a bigger association between STIM1 and Orai1 in thrombotic patients (see Figure 5). Conversely, STIM1 /TRPC1 coupling did not significantly change, despite an increased tendency being observed in some thrombotic patients. We also investigated possible downregulation of SARAF expression, which may increase SOCE activation in platelets [54,55], but we did not find significant modification between healthy and thrombotic platelets. Platelets from STC2 KO mice presented normal SOCE, but enhanced expression of non-capacitative Ca^2+^ entry was found instead. Particularly, increased Orai3 was observed in the murine STC2 KO platelets. Conversely, in platelet samples drawn from thrombotic patients, we found reduced TRPC6 and Orai3 [20]. This discrepancy could be explained by the fact that murine platelets completely lack STC2 expression due to the mice being STC2 KO; meanwhile, thrombotic patients presented reduced STC2 expression. STC2 is still present and, therefore, retains a regulatory role on SOCE in these platelets (Figure 7). Hence, platelets from thrombotic patients would not require an additional source of Ca^2+^ to develop their role in hemostasis.

## 4. Materials and Methods

### 4.1. Patients: Healthy Subjects and Mice

Forty-one thrombotic patients, mostly from Western Europe and the Caucasian subpopulation, with an age range of 51 ± 10 (S. D.) years old, were enrolled in the present study upon accepting the respective informed consent; patients presented a similar gender distribution. B.N. selected the patients during their first visit to the hematology specialist, and B.N. considered the following inclusion criteria: being over 18 years old, clinical history of being diagnosed as thrombotic patients, and, finally, their adherence to medication administered from the time they were discharged from the emergency unit of San Pedro de Alcántara Hospital. An initial cross-sectional study was performed to identify a possible alteration in the expression of STCs in these patients compared to healthy volunteers. Following that, we performed a second round of blood extractions 10 years later on patients who showed alterations in the STC2 expression values. This longitudinal study’s inclusion criteria were that the patients had suffered a stroke/ictus, remained adherent to the initial prescribed medication (mainly ASA 100 mg daily), and, further, they must not present a recurrent thrombotic episode; otherwise, patients were excluded from the study (just 8 patients met those criteria and finalized the study). Nonetheless, STC2 values from patients who did not satisfy these inclusion criteria were only considered for the initial cross-sectional analysis. A total of 15 healthy subjects were recruited from the volunteers who regularly attended the blood bank of Extremadura; among them, we selected volunteers who met the following criteria: a similar age range as thrombotic patients, as well as a similar gender distribution. These volunteers were also asked to complete the informed consent, and we previously confirmed that they had not suffered a previous thrombotic incident or were not medicated. These informative consents were kept in custody by hematologists of either the San Pedro de Alcántara Hospital or the Blood Bank of Extremadura.

Alternatively, we performed an antibody specificity test using murine platelets from WT and STC2 KO mice. The mice were kindly provided by Prof. Roger Reddel from the University of Sydney (Australia), maintained under regular breeding conditions, and regularly bled from the retro-orbital plexus. The protocols performed on animals agree with the bioethical regulation guidelines, and we obtained permission from the local animal committee of the University of Extremadura. Blood was used as a surrogate for human blood, and upon isolating platelets from WT and STC2 KO mice, WB was performed as detailed in the following sections to ensure antibody specificity according to the predicted bands at the right molecular mass for STC2 (around 33 kDa), as shown in Supplementary Figure S1C.

### 4.2. Blood Sampling and Conservation

Blood was drawn from thrombotic patients and healthy volunteers immediately after being enrolled in the research project by our hematologist during their first visit to this unit. Blood extraction was performed via venipuncture from the cubital vein by qualified personnel using sterile materials. Samples were immediately mixed with citrate sterile tubes and were maintained at room temperature (depending on the environmental temperature), kept under constant agitation, and anonymized using a numeric code; thus, physicians were the only ones able to identify the blood donors to obtain their clinical histories for subsequent correlation analysis (see Supplementary Table S1, where clinical and demographic variables are presented). Blood samples were immediately delivered to the Department of Physiology of the University of Extremadura within hours following the extraction or were alternatively conserved under constant agitation and temperature-conditioned to around 30–37 °C. Blood samples received within the following 12h after their extractions were considered suitable for the present study; otherwise, the blood was discarded. The local ethical committee guidelines of the University of Extremadura and the Ethical Committee for Clinical Research of Extremadura County Health Service (resolution positive on 27 January 2015) were taken into consideration during the experiment design, and the protocols were also in accordance with the Declaration of Helsinki.

### 4.3. Cell Culture and Glycosylation Analysis of STC2

Protein glycosylation, together with ubiquitination and phosphorylation, may affect the protein molecular weight and thus result in unexpected banding when analyzed by WB. STC2 has been reported to be glycosylated and phosphorylated as part of its post-translational modifications. Therefore, we confirmed the STC2 glycosylation by incubating human platelet lysates obtained from healthy subjects as well as cell lysates from MEG-01 (a widely used platelet progenitor cell) and HEK293 cells (non-related to the platelet cell lineage) with PNGase F overnight at 37 °C before protein denaturation by mixing with LB (5% DTT). Subsequent WB was completed with an anti-STC2 antibody; the images are shown in Supplementary Figure S1D.

MEG-01 and HEK293 cells were ordered from ATCC^®^ collection (Manassas, VA, USA) and were maintained following supplier recommendations (37 °C, 5% CO_2_, and 95% humidity) using either RPMI or DMEM, respectively. Finally, the medium was replaced every two days and before cells reached over 80% confluence to ensure an optimal survival rate. STC2 silencing in MEG-01 cells was achieved by treating the cells with a mixture of lipofectamine ^®^ 3000 (Thermo Fisher Scientific, Waltham, MA, USA) and mission^®^esiRNA STC2 (catalogue number: EHU029291 from Sigma-Aldrich, Madrid, Spain), and following that, they were cultured for 72h. Silencing efficiency was then corroborated by WB using an anti-STC2 antibody (clone 2B11; Sigma-Aldrich^®^, Madrid, Spain #WH0008614M8).

### 4.4. Preparation of Platelet Protein Samples for Immunoprecipitation and Western Blotting

Blood samples were subjected to differential centrifugation (700× *g* for 5 min), which resulted in the differentiation of three main blood phases: PRP (on top of the test tube), leukocytes (interphase), and erythrocyte pelleted phase (bottom of the test tube). The PRP was aspirated with a Pasteur pipette, leaving a small amount of the PRP (5–10 mm just above erythrocytes-PRP interphase), and thus we ensured that we avoided contamination of the PRP with erythrocytes and leukocytes (the latter also confirmed by observing the images obtained with bright-field microscopy). The PRP was then supplemented with apyrase (40 μM) and ASA (100 μM) to avoid platelet activation because of the mechanical stress evoked during the platelet isolation protocol. PRP was subsequently centrifuged at 350× *g* (for 20 min) to obtain Platelet-Poor Plasma (PPP) and the platelet pellets at the bottom of the test tube. Finally, after removing the PPP from the platelet pellets, they were immediately mixed with Laemmli’s buffer (pH 6.8, containing 0.62 M Tris–Cl and 2% SDS, 10% glycerol, 0.002% bromophenol blue, and under reducing conditions by adding 5% of dithiothreitol was added) in the case of WB or with NP40 buffer (pH 8, containing 137 mM NaCl, 20 mM Tris, 2 mM EDTA, and supplemented with 10% glycerol, 1% Nonidet P-40, and Na_3_VO_4_ and protease cocktail) in the case of being required in a previous step during the immunoprecipitation protocol, which was followed by cell fixation with Laemmli’s buffer to perform the subsequent WB. Crosslinking reagents were not used during the IP protocol.

The immunoprecipitation protocol consisted of incubating the proteins isolated from the platelet samples overnight at 4 °C and under constant rotation with 2 μg/mL of a specific anti-STC2 or anti-STIM1/GOK (Clone 44; BD Biosciences, Madrid, Spain #610954) primary antibodies and a bead of agarose. The next day, immunoprecipitated samples were subjected to a washing steps that consisted of six centrifugations (10,000× *g* for 5 min), followed by subsequent resuspension in fresh PBS solution, which avoided recognition of the non-specific binding sites by the antibody used for immunoprecipitation of the protein complexes of interest and, following, these immunoprecipitated protein complexes were mixed with Laemmli’s buffer (5% DTT) and subjected to physical denaturalization at 95 °C for 5 min, upon which protein samples were frozen at −80 °C until required.

WB was performed to visualize the proteins of interest using SDS-page 10% acrylamide–acrylamide gels that were loaded with around 50 μg of protein in each gel lane, after which proteins were subsequently electrotransferred for 2 h onto nitrocellulose membranes using a semidry blot device. Finally, the membranes were exposed to the respective primary antibodies using the following exposure times and adequate concentrations. Thus, we used monoclonal anti-STC1 (Sigma-Aldrich^®^, Madrid, Spain # SAB2102323) and anti-STC2 (clone 2B11) antibodies for 2 h that were diluted 1:1000 in Tris-buffered saline with Tween^®^ 20 Detergent (TBST); monoclonal anti-phosphor-serine (clone 4A4; Millipore^®,^ Madrid, Spain #05-1000) antibody incubated for 1h (1:5000 in TBST); polyclonal anti-TMEM66 (SARAF) antibody (Thermo-Fisher, Madrid, Spain #PA5-24237) for 1 h. (1:1000 in TBST); monoclonal anti-Orai1 antibody (Sigma-Aldrich^®^, Madrid, Spain #08264) for 2 h (1:1000 in TBST); monoclonal anti-Orai3 antibody (Clone 1B4F1; Sigma-Aldrich^®^, Madrid, Spain #SAB3500121) overnight (1:250 in TBST); polyclonal anti-TRPC1 antibody (Origene^®^, Rockville, MD, USA # SKU TA328677) for 2 h (1:1000 in TBST); polyclonal anti-TRPC3 antibody (Abcam^®^, Cambridge, UK #ab51560) overnight (1:1000 in TBST); polyclonal anti-TRPC6 antibody (Alomone Labs Ltd. Jerusalem, Israel #ACC-017); monoclonal anti-STIM1/GOK primary (Clone 44; BD biosciences, Madrid, Spain #610954) antibody for 1 h (1:1000 in TBST).

Finally, after washing six times for 5 min using fresh TBST, the membranes were exposed for 1h to the appropriate secondary antibodies (previously diluted 1:5000 or 1:10,000 as required). The membranes were finally exposed to enhanced HRP-ECL pico-Plus (Thermo-Fisher scientific^®^, Madrid, Spain # 15513766) for 5 min, and densitometry images were obtained with a Biorad^®^-ChemiDoc imaging system (Madrid, Spain). These images were analyzed using free Fiji v1.54P software from NIH, USA.

Additionally, the membranes were reprobed either with the respective antibody used for immunoprecipitation of the protein complexes or with an anti-actin (clone AC-15; Sigma-Aldrich^®^ #A5441) antibody for 1 h (diluted 1:1000 in TBST), with these results used as a protein loading control. This means that before comparing the different results from each group, the data of the target protein was previously normalized by considering its ratio with respect to the amount of β-actin that was loaded in each gel lane.

### 4.5. ”In Silico” Analysis of the Proteins

Two main databases and “in silico” analysis were used in this study. Initially, we used the protein database from NIH through PUBMED to obtain a FASTA sequence of the protein, which was then used to confirm the actual configuration of the 3D reconstruction. The 3D structures of STC1, STC2, and STC2 complexed with pappalisin A1 (PAP-A1) were generated using free AlphaFold^®^ software (https://alphafold.ebi.ac.uk; accessed on 1 May 2025), which allows us to modify the protein images with the residues of interest by changing the design and color of these relevant residues. Furthermore, in the AlphaFold^®^ database, structures of proteins are also available in combination with toxins and cations bound to their target residues. Both types of tools were used to generate the images included in the present study. Additionally, we used NetPhos 3.1 (https://services.healthtech.dtu.dk/services/NetPhos--3.1/; accessed on 1 May 2025) to predict the phosphorylation status of STC2, including the possible kinases that are actually identified and may be suitable to phosphorylate the STC2 residues at tyrosine, serine, and threonine residues, respectively. The latter makes it possible for us to evaluate the possible serine phosphorylation of STC2 presented in the present study.

### 4.6. Ca^2+^ Homeostasis Evaluation

MEG-01 cells were transfected using lipofectamin^®^ 3000 and siRNA A or SiRNA STC2, and after 72h, cell samples (10^6^ cell/mL) were fixed with LB (5%) for WB analysis using anti-STC2 antibody to demonstrate silencing efficiency; meanwhile, the rest of the cells from the same culture dish were incubated for 30 min at r.t. with the calcium dye fura-2 AM. Following this, the cells were allowed to adhere to coverslips coated with poly-L-lysine, and they were placed into a perfusion chamber. The culture medium was replaced with HEPES buffer saline supplemented with 50 μM of CaCl_2_ to avoid depletion of the intracellular stores due to cell manipulation and to also remove the excess Ca^2+^ dye. The cells were alternatively excited at a 340/380 nm wavelength, and the fura-2 fluorescence emitted by the cells at 505 nm was recorded using an inverted fluorescence microscope (Nikon^®,^ Nikon Eclipse Ti2, Amsterdam, The Netherlands; this fluorescence was recorded with a CCD camera (Hisca CCD C-6790, Hamamatsu, Japan) with Aquacosmos 2.5 software implemented (Hamamatsu Photonics, Hamamatsu, Japan). The analysis of the changes in Ca^2+^ homeostasis was performed by adding EGTA (75 μM) to cells at the beginning of each experiment to remove the extracellular Ca^2+^. Following this, the cells were incubated for 4 min with 1 mM of TG to evoke Ca^2+^ store depletion, and the subsequent activation of SOCE was monitored for 2 min by adding 1 mM of CaCl_2_ to the extracellular medium. The changes in [Ca^2+^]_i_ were represented by considering changes in fura2 fluorescence (with F_n_/F_0;_ indicating the F_0_ fura-2 fluorescence ratio after adding EGTA) and comparing between different groups by estimating the areas under curves after the addition of the TG and CaCl_2_, respectively, which was represented as the mean ± S.E.M of the percentage with respect to cells transfected with SiRNA A.

### 4.7. Statistical Analysis

Data was analyzed and represented using GraphPad Prism v 9.0 software. The Mann–Whitney U-test was used to compare data that was not normally distributed; a 95% confidence level was considered here. Statistical significance between groups was established by using the non-parametric Kruskal–Wallis test, followed by Dunn’s post-test, which was used for comparison of protein expression values between different pharmacological treatments. *p* < 0.05 was considered significant.

## 5. Conclusions

STC2 controls SOCE in platelets and other cell types. Silencing of STC2 increased SOCE in the platelet lineage as a result of increasing the STIM1/Orai1 association. Because Ca^2+^ entry into platelets is crucial for platelet aggregation, and we found downregulation of STC2 expression in the platelets from thrombotic patients, STC2 alteration may underlie thrombotic incidents in these subjects, particularly in thrombotic patients suffering from stroke/ictus. Future multicenter analysis, as well as additional studies searching for drugs that could target STC2, are required to finally consider STC2 as an efficient marker of thrombotic accidents.

## Data Availability

The original contributions presented in this study are included in the article/Supplementary Material. Further inquiries can be directed to the corresponding authors.

## Supplementary Materials

The following supporting information can be downloaded at: https://www.mdpi.com/article/10.3390/ijms26209999/s1.

## Abbreviations

The following abbreviations are used in this manuscript:

STC	Stanniocalcin
ASA	Acetyl salicylic acid
STIM	Stromal interaction molecule
Orai	ORAI calcium release-activated calcium modulator
SOCE	Store-operated Ca^2+^ entry
TRPC	Transient receptor potential channel
[Ca^2+^]_i_	Intracellular calcium concentration
SARAF	Store-operated calcium entry-associated regulatory factor (TMEM66)
PAPP-A	Pregnancy associated plasma protein-A
LMWH	Low-molecular-weight heparin
PKA	Protein kinase A
PKC	Protein kinase C
Ser	Serine
Thr	Threonine
Tyr	Tyrosine
HIF-1	Hypoxia-induced factor 1
PRP	Platelet-rich plasma
PPP	Platelet-poor plasma
TBST	Tris-buffered saline with Tween^®^ 20 Detergent
