# Supplementary material for: Stanniocalcin2, A Promising New Target for Identifying Patients with Stroke/Ictus
Source: Int J Mol Sci. 2025 Oct 14;26(20):9999. doi: 10.3390/ijms26209999 (PMC12564801; doi:10.3390/ijms26209999)
Supplement: Supplementary file 1 [file ijms-26-09999-s001.zip › ijms-3800895-Table S1.pdf]

| | Demographic | | | | Clinical charasterististic | |
|---|---|---|---|---|---|---|
| | gender (male) | age (years) | Smoker | Smoker history | Diagnosed | Comorbidites |
| # 1 | Male | 45 | 1 | leave 10 years | Myocardial infartion | Hepatic steatosis; hyperglycemia (134 mg/dL) |
| # 2 | Female | 31 | 0 | 0 | Stroke | Hypercholesterolemia; normoglycaemia (88 mg/dL] |
| # 3 | Female | 52 | 0 | 0 | Cryptogenic stroke | Hypoglycaemia (77) |
| # 4 | Female | 44 | 1 | 0 | Myocardial infartion | Hypercholesterolemia; hypoglycaemia (77 mg/dL) |
| # 5 | Female | 57 | 0 | 0 | Brain Stroke | Normoglycaemia (90 mg/dL); renal disease; low platelet account |
| # 6 | Female | 66 | 0 | 0 | Brain Stroke | Normoglycaemia (89 mg/dL); low platelet account; hypothyrodism;BMI 24.8 |
| # 7 | Male | 46 | 0 | 0 | Deep venous thrombosis | Hypoglycaemia (77); abdmonial surgery; low homoscistein |
| # 8 | Male | 42 | 0 | 0 | Antiphophol. Syndr. heart stroke | Normoglycaemia (104 mg/dL) |
| # 9 | Female | 40 | 1 | leave 1 year | Brain Stroke | Normoglycaemia (101 mg/dL); low platelet account; BMI 33.71 |
| # 10 | Female | 46 | 1 | | Brain Stroke | Normoglycaemia (86 mg/dL); low platelet account |
| # 11 | Female | 34 | 0 | 0 | Brain Stroke | Normoglycaemia (99 mg/dL) |
| # 12 | Female | 65 | 1 | leave 10 years | Brain Stroke | Normoglycaemia (95 mg/dL) |
| # 13 | Male | 59 | 0 | 0 | Brain Stroke | Normoglycaemia (95 mg/dL) |
| # 14 | Male | 56 | 1 | leave 10 years | Deep venous thrombosis | Normoglycaemia (92 mg/dL) |
| # 15 | Female | 45 | 1 | 0 | Brain Stroke | Hypoglycaemia (73 mg/dL) |
| # 16 | Female | 47 | 0 | 0 | Brain Stroke | Normoglycaemia (95 mg/dL) |
| # 17 | Female | 48 | 0 | 0 | Lung Venous thrombosis | Normoglycaemia (92 mg/dL); beta-lactamic alergy |
| # 18 | Female | 85 | 0 | 0 | Lung Venous thrombosis | Normoglycaemia (104 mg/dL); hypercholesterolemia; Paget disease |
| # 19 | Female | 59 | 0 | 0 | N.R. | Normoglycaemia (94 mg/dL) |
| # 20 | Male | 60 | 1 | 0 | Brain Stroke | Hypercholesterolemia; Low platelet count; normoglycaemia (83 mg/dL);BMI 26 |
| # 21 | Male | 62 | 0 | leave 20 years | Brain Stroke | Normoglycaemia (94 mg/dL); Mutación FII |
| # 22 | Female | 41 | 0 | leave 1 year | Brain Stroke | Normoglycaemia (91 mg/dL);Hyperthyridism |
| # 23 | Female | 55 | 0 | 0 | Brain Stroke | Normoglycaemia (97 mg/dL) |
| # 24 | Male | 50 | 1 | 0 | Deep venous thrombosis | Bone traumatism |
| # 25 | Male | 45 | 1 | 0 | Stroke | Normoglycaemia (94 mg/dL); alcoholic |
| # 26 | Male | 45 | 1 | | Stroke | Normoglycaemia (95 mg/dL) |
| # 27 | Female | 27 | 0 | 0 | Deep venous thrombosis | Normoglycaemia (85 mg/dL) |
| # 28 | Female | 56 | 0 | 0 | Stroke | Normoglycaemia (94 mg/dL) |
| # 29 | Male | 52 | 1 | leave 1 year | Stroke | Normoglycaemia (93 mg/dL); BMI 31 |
| # 30 | Male | 59 | 0 | 0 | Stroke | Normoglycaemia (99 mg/dL) |
| # 31 | Male | 57 | 0 | 0 | Stroke | Normoglycaemia (93 mg/dL) |
| # 32 | Male | 53 | 0 | 0 | Stroke | Hyperglycaemia (111 mg/dL); BMI 29 |
| # 33 | Female | 70 | 0 | 0 | Brain Stroke | Normoglycaemia (94 mg/dL); low platelet account; BMI 22 |
| # 34 | Female | 47 | 0 | 0 | Stroke | Normoglycaemia (85 mg/dL) |
| # 35 | Male | 51 | 1 | Leave 1 year | Myocardial infartion | Normoglycaemia (106 mg/dL) |
| # 36 | Male | 47 | 0 | 0 | Deep venous thrombosis | Normoglycaemia (92 mg/dL); low platelet account |
| # 37 | Male | 40 | 1 | leave 1 year | Stroke | Normoglycaemia (83 mg/dL) |
| # 38 | Male | 55 | 0 | 0 | Deep venous thrombosis | Hyperuricemia |
| # 39 | Male | 51 | 1 | leave less 1 year | Stroke | N.R. |
| # 40 | Male | 41 | 1 | leave less 1 year | Stroke | Sporadic drug consumer |
| # 41 | Male | 59 | 1 | Leave 1 year | Stroke | Gastric ulcer; hyperhomeocysteinemia |

| | Biochemical analyses | | | | | Coagulation features | | | | | | | |
|---|---|---|---|---|---|---|---|---|---|---|---|---|---|
| | Creatine (µM) | Cholesterol (mM) | LDL-C (mM) | HDL-C (mM) | triglycerides (mM) | Prothrombin time ratio | Prothrombin index | INR | Fibrinogen | Anti-thrombin Atb. | Protein S | Lupic antigen | Protein C |
| # 1 | N.R. | N.R. | N.R. | N.R. | N.R. | N.R. | | | | | | | |
| # 2 | N.R. | N.R. | N.R. | N.R. | N.R. | 0.85 | 9.5 | 0.85 | 3.11 | 101 | 112.8 | Negative | 121 |
| # 3 | N.R. | N.R. | N.R. | N.R. | N.R. | N.R. | N.R. | N.R. | N.R. | 111 | 66.7 | Negative | 90 |
| # 4 | 0.55 | 189 | 104 | 54 | 154 | N.R. | N.R. | N.R. | N.R. | 117 | 60.7 | Negative | 103 |
| # 5 | N.R. | N.R. | N.R. | N.R. | N.R. | N.R. | N.R. | N.R. | N.R. | 144 | 52.4 | Negative | 78 |
| # 6 | N.R. | N.R. | N.R. | N.R. | N.R. | N.R. | N.R. | N.R. | N.R. | 90 | 68.4 | Negative | 88 |
| # 7 | N.R. | N.R. | N.R. | N.R. | N.R. | N.R. | N.R. | N.R. | N.R. | 94 | 90.2 | Negative | 112 |
| # 8 | N.R. | N.R. | N.R. | N.R. | N.R. | N.R. | N.R. | N.R. | N.R. | 127 | 104 | Negative | 84 |
| # 9 | N.R. | N.R. | N.R. | N.R. | N.R. | 0.99 | 11.1 | 0.99 | 4.07 | 103 | 83.1 | Negative | 81 |
| # 10 | 0.97 | 206 | N.R. | N.R. | 155 | 2.56 | 28.7 | 2.54 | 4.75 | 110 | 61.7 | Positive | 76 |
| # 11 | N.R. | N.R. | N.R. | N.R. | N.R. | N.R. | N.R. | N.R. | N.R. | 107 | 95 | Negative | 103 |
| # 12 | 0.75 | 171 | 80 | 77 | 68 | 1.03 | 11.5 | 1.02 | 4.56 | 97 | 106 | Negative | 94 |
| # 13 | N.R. | N.R. | N.R. | N.R. | N.R. | 0.93 | 10.4 | 1.2 | 3.51 | 100 | 95.5 | Negative | 103 |
| # 14 | N.R. | N.R. | N.R. | N.R. | N.R. | 0.88 | 9.9 | 0.96 | 3.62 | 124 | 106.7 | Negative | 152 |
| # 15 | 0.77 | 242 | 146 | 82 | 72 | 0.88 | 9.8 | 0.87 | 4.17 | 97 | 112.1 | Negative | 129 |
| # 16 | 0.62 | 151 | N.R. | N.R. | 51 | 1.05 | 11.8 | 1.05 | 3.92 | 35 | 62.8 | Negative | 75 |
| # 17 | N.R. | N.R. | N.R. | N.R. | N.R. | N.R. | N.R. | N.R. | N.R. | 191 | N.R. | Negative | N.R. |
| # 18 | N.R. | N.R. | N.R. | N.R. | N.R. | N.R. | N.R. | N.R. | N.R. | 178 | 57.4 | Negative | 116 |
| # 19 | N.R. | N.R. | N.R. | N.R. | N.R. | N.R. | N.R. | N.R. | N.R. | N.R. | N.R. | N.R. | N.R. |
| # 20 | N.R. | N.R. | N.R. | N.R. | N.R. | N.R. | N.R. | N.R. | N.R. | N.R. | 113 | Negative | N.R. |
| # 21 | 0.89 | 120 | 397 | N.R. | 49 | N.R. | N.R. | N.R. | N.R. | 116 | 61 | Negative | 88 |
| # 22 | 0.72 | 127 | 59 | N.R. | 127 | 1.01 | 11.3 | 1.01 | 4.12 | 108 | 57.4 | Negative | 71 |
| # 23 | N.R. | N.R. | N.R. | N.R. | N.R. | 0.94 | 10.5 | 0.94 | 4.27 | 116 | 81.8 | Positive | 93 |
| # 24 | N.R. | N.R. | N.R. | N.R. | N.R. | N.R. | N.R. | N.R. | N.R. | 186 | 74 | Negative | 118 |
| # 25 | 0.7 | N.R. | N.R. | N.R. | N.R. | 0.94 | 10.5 | 0.96 | 6.5 | 92 | 89 | Negative | 89 |
| # 26 | N.R. | N.R. | N.R. | N.R. | N.R. | N.R. | N.R. | N.R. | N.R. | N.R. | N.R. | Negative | |
| # 27 | N.R. | N.R. | N.R. | N.R. | N.R. | N.R. | N.R. | N.R. | N.R. | N.R. | N.R. | N.R. | N.R. |
| # 28 | N.R. | N.R. | N.R. | N.R. | N.R. | N.R. | N.R. | N.R. | N.R. | 116 | 81.8 | Positive | 793 |
| # 29 | N.R. | N.R. | N.R. | N.R. | N.R. | 1.13 | 12.6 | 1.13 | 4.67 | 128 | 74.3 | Negative | 98 |
| # 30 | N.R. | N.R. | N.R. | N.R. | N.R. | N.R. | N.R. | N.R. | N.R. | N.R. | N.R. | N.R. | N.R. |
| # 31 | N.R. | N.R. | N.R. | N.R. | N.R. | N.R. | N.R. | N.R. | N.R. | 101 | 110.2 | Negative | 106 |
| # 32 | N.R. | N.R. | N.R. | N.R. | N.R. | N.R. | N.R. | N.R. | N.R. | N.R. | N.R. | N.R. | N.R. |
| # 33 | N.R. | N.R. | N.R. | N.R. | N.R. | N.R. | N.R. | N.R. | N.R. | N.R. | N.R. | N.R. | 23 |
| # 34 | N.R. | N.R. | N.R. | N.R. | N.R. | N.R. | N.R. | N.R. | N.R. | N.R. | N.R. | N.R. | N.R. |
| # 35 | N.R. | N.R. | N.R. | N.R. | N.R. | N.R. | N.R. | N.R. | N.R. | N.R. | N.R. | N.R. | N.R. |
| # 36 | N.R. | N.R. | N.R. | N.R. | N.R. | N.R. | N.R. | N.R. | N.R. | N.R. | 48 | Positive | N.R. |
| # 37 | N.R. | N.R. | N.R. | N.R. | N.R. | N.R. | N.R. | N.R. | N.R. | 115 | 91.4 | Negative | 86 |
| # 38 | N.R. | N.R. | N.R. | N.R. | N.R. | N.R. | N.R. | N.R. | N.R. | 87 | 88 | Negative | 109 |
| # 39 | N.R. | N.R. | N.R. | N.R. | N.R. | 0.85 | 11.3 | 1.01 | 5.52 | 100 | 100.3 | Positive | 84 |
| # 40 | N.R. | N.R. | N.R. | N.R. | N.R. | 1.04 | 12 | 1.07 | 3.35 | 91 | 93.1 | Negative | 86 |
| # 41 | N.R. | N.R. | N.R. | N.R. | N.R. | 0.91 | 10.2 | 0.91 | 3.81 | 114 | 76 | Negative | 73 |

| | Antithrombotic drugs | | | | Other drugs history |
|---|---|---|---|---|---|
| | ASA | Clopidogrel | LMWH | Others | |
| # 1 | NO | NO | NO | NO | atorvastatin, clopidogrel, ursodeoxicolic acid, pantoprazole, ramipril, zolpidem |
| # 2 | Yes | NO | NO | NO | bisoprolol, lorazepam, nitroglicerin, omeprazole, rosuvastatin, ticagrelor |
| # 3 | NO | NO | NO | Yes | acetaminophen, acenocoumarol, eutirox, naloxone, oxycodone, pregabalin |
| # 4 | NO | NO | Yes | NO | atorvastatin, calcium, colecalciferol, diazepam, lormetazepam, metamizole, mianserina, ranitidin, |
| # 5 | NO | NO | Yes | NO | atorvastatin, metamizole, olmersatan, pantoprazole |
| # 6 | Yes | NO | NO | NO | acenocoumarol, diazepam,irbersartan, biperiden, loratadina, omeoprazole, risperidone |
| # 7 | Yes | NO | NO | NO | amitriptilin, atorvastatin; omeprazole, sertralin, sulpiride |
| # 8 | Yes | NO | NO | NO | atorvatatin, baclofen, clomipromine, diazepam, omeprazole |
| # 9 | Yes | NO | NO | NO | rosuvastatin |
| # 10 | Yes | NO | NO | Yes | acetaminophen, atorvastatin, citicolin, escitalopram, lormetazepam, metamizole, tiamazol,topiramate, omeprazole, piracetam |
| # 11 | NO | NO | NO | NO | N.R. |
| # 12 | Yes | NO | NO | NO | acetaminophen; atorvastatin; omeprazole |
| # 13 | Yes | NO | NO | NO | atorvastatin; betahistine; enalapril; fluoxetin; metamizole; ursodeoxicolic acid |
| # 14 | Yes | NO | NO | NO | N.R. |
| # 15 | Yes | NO | NO | NO | atorvastatin; omeprazol |
| # 16 | NO | Yes | NO | NO | acetaminophen, levetirazepam; metamizole; ranitidine; rotigotine; |
| # 17 | NO | NO | NO | NO | acenocoumarol, dexketoprofen, iron |
| # 18 | NO | NO | Yes | NO | metamizole, olmesartan, pantoprazole, simvastatin |
| # 19 | N.R. | N.R. | N.R. | N.R. | N.R. |
| # 20 | Yes | NO | NO | NO | atorvastatin; bisoprolol; pantoprazol; ramipril |
| # 21 | Yes | NO | NO | NO | amitriptilina, atorvastatin, omeprazol, sertraline, sulpiride |
| # 22 | Yes | NO | NO | NO | atorvastatin, baclofen, clomipramine, omeprazole, diazepam |
| # 23 | N.R. | N.R. | N.R. | N.R. | N.R. |
| # 24 | NO | NO | NO | Yes | acenocoumarol, bromazepam |
| # 25 | NO | NO | NO | NO | enalapril |
| # 26 | Yes | NO | NO | NO | omeprazole |
| # 27 | NO | NO | Yes | NO | N.R. |
| # 28 | Yes | NO | NO | NO | acetaminophen, atorvastatin, escitalopram, lormetazepam, metamizole, omeprazole, piracetam, thiamazole |
| # 29 | Yes | NO | NO | NO | atorvastatin |
| # 30 | NO | NO | Yes | NO | acetaminophen, atenolol, atorvastatin, donepezil, doxazosine, furosemide, risperidone, zolpidem |
| # 31 | Yes | NO | NO | NO | atorvastatin, omeprazole, perindropil |
| # 32 | Yes | NO | NO | NO | amitriptyline,escitalopram, etoricoxib |
| # 33 | NO | Yes | NO | NO | levetirazepam, ranitidine, rotigotine |
| # 34 | Yes | NO | NO | NO | amlodipine, atorvastatin, clorazepate, cyproterone, ethinyl estradiol, fluoxetine, gabapentin, metamizole, mirtazapine, omeprazole; ramipril, |
| # 35 | Yes | NO | NO | NO | atorvastatin,bisoprolo ,pantoprazole, ramipril |
| # 36 | NO | NO | NO | Yes | acenocoumarol, calcipotriol/betametasone (topic) |
| # 37 | Yes | NO | NO | NO | atorvastatin, omeprazole |
| # 38 | N.R. | N.R. | N.R. | N.R. | N.R. |
| # 39 | Yes | NO | NO | NO | N.R. |
| # 40 | Yes | NO | NO | NO | N.R. |
| # 41 | Yes | NO | NO | NO | omeprazole |

* N.R.: non reported

| Gender | Age average | | Smoker habits |
|---|---|---|---|
| 21 M (51 %) | 51.0 | Mean | 17 (41%) |
| 20 F (49 %) | 10.8 | S.E.M. | |

| Clinical charasterististic | |
|---|---|
| Main diagnosed illnesses | Comorbidities |
| Stroke | Obesity |
| Deep venous thrombosis | Hypercholesterolemia |
| | Genetic mutation associated to thrombotic illness |

| | Coagulation features | | | | | | | |
|---|---|---|---|---|---|---|---|---|
| | Prothrombin time ratio | Prothrombin index | INR | Fibrinogen | Anti-thrombin Atv. | Protein S | Lupic antigen | Protein C |
| Mean | 1.1 | 12.1 | 1.1 | 4.3 | 113.2 | 83.1 | | 117.4 |
| S.E.M. | 0.4 | 4.7 | 0.4 | 0.9 | 30.6 | 19.8 | | 129.6 |

| Antithrombotic drugs | | | |
|---|---|---|---|
| ASA | Clopidogrel | Heparine | Others |
| 24 (58%) | 2 (5%) | 5 (12%) | 4 (10 %) |
| | | | |
