# Supplementary figures and images for: Stanniocalcin2, A Promising New Target for Identifying Patients with Stroke/Ictus
Source: Int J Mol Sci. 2025 Oct 14;26(20):9999. doi: 10.3390/ijms26209999 (PMC12564801; doi:10.3390/ijms26209999)

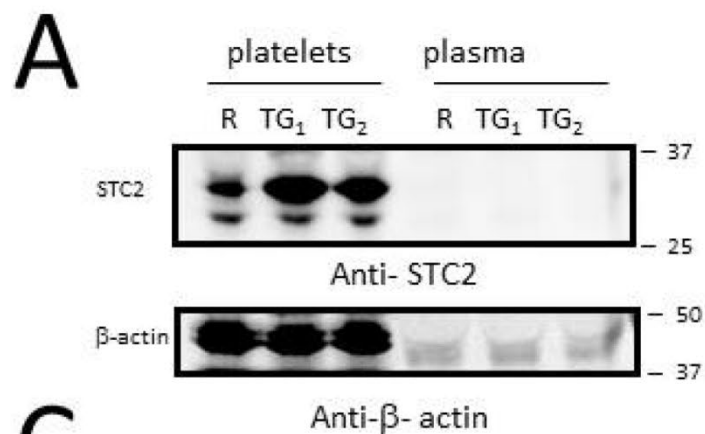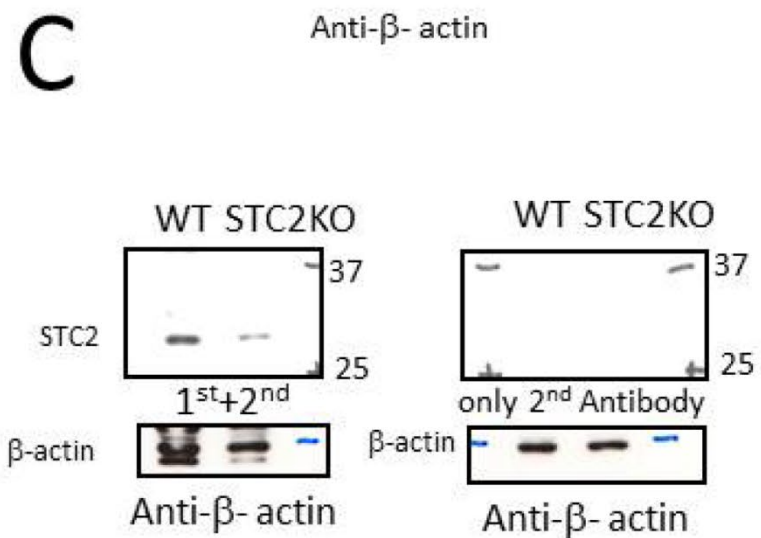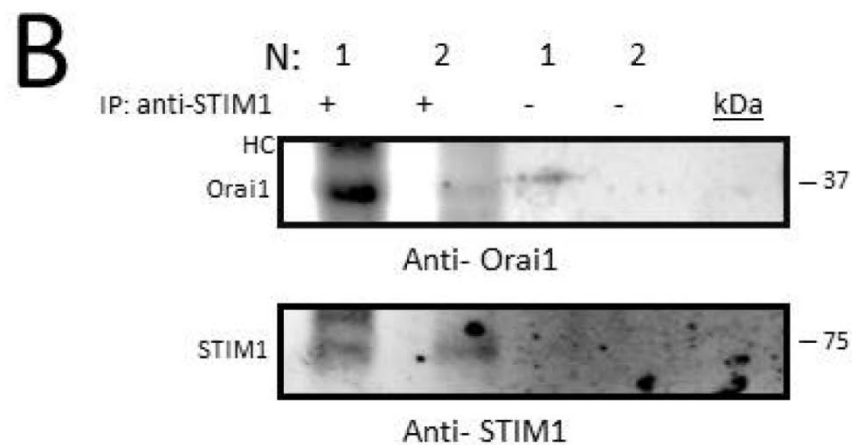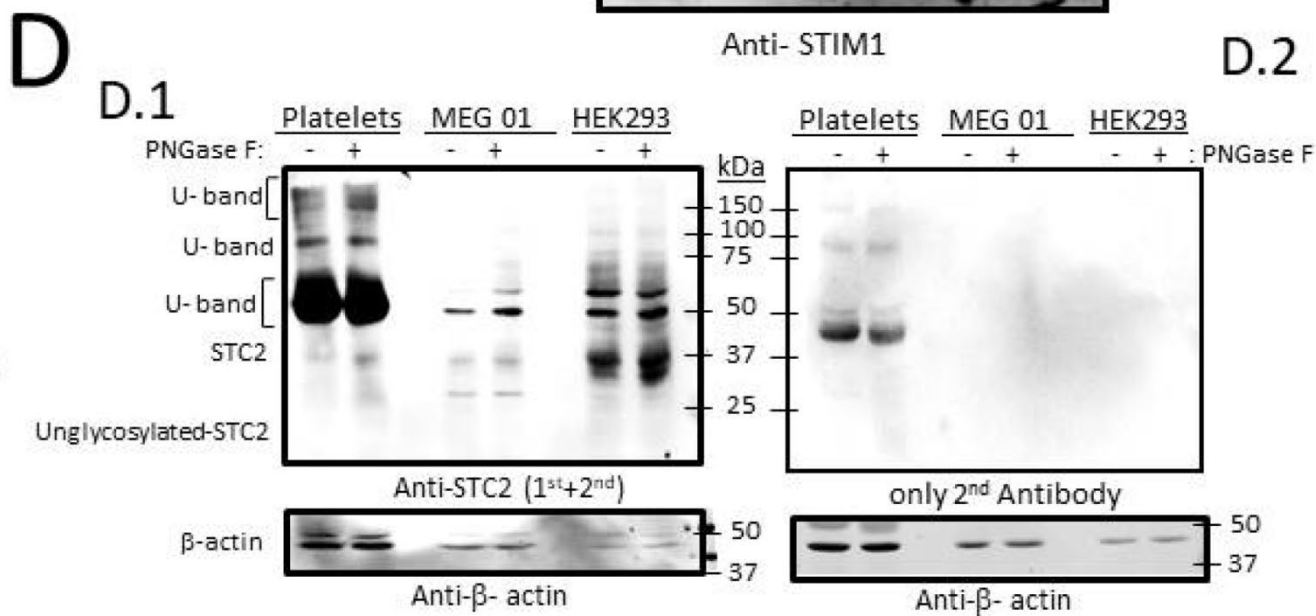

Supplement: Supplementary file 1 [file ijms-26-09999-s001.zip › ijms-3800895-Supplementary Figure S1.pdf]
